# An analysis of disparities in imaging for suspected traumatic brain injury among patient visits to emergency departments (2010–2021)

**DOI:** 10.3389/fneur.2025.1621761

**Published:** 2025-09-11

**Authors:** Susan Avila Misciagno, Erin Dancy, Michael R. Jiroutek, Melissa A. Holland

**Affiliations:** Department of Pharmaceutical and Clinical Sciences, Campbell University, Buies Creek, NC, United States

**Keywords:** brain injury, brain trauma, healthcare survey, hospital emergency services, diagnostic imaging, healthcare disparities

## Abstract

**Background:**

Traumatic brain injuries (TBI) are an increasing global public health concern. Current guidelines for neuroimaging lack sufficient evidence on specific recommendations for conducting diagnostic imaging during emergency department (ED) visits for TBI. Moreover, imaging underutilization and healthcare disparities persist despite evidence that early access to imaging can significantly impact the prediction of functional outcomes in patients presenting with suspected TBI. Therefore, investigating neuroimaging utilization nationwide for patient screening amongst those who present to the ED for TBI is crucial.

**Methods:**

This retrospective, observational, cross-sectional study was conducted using data from the National Hospital Ambulatory Medical Care Survey (NHAMCS), an annual, nationally representative sample survey of visits to hospital EDs in the United States, conducted by the National Center for Health Statistics. A multivariable logistic regression model was constructed to evaluate predictors of interest available in the database on the use of imaging for visits to the ED with a suspected TBI. A graph of the annual percentage of ED visits at which imaging was used was constructed.

**Results:**

Visits by the Black race group compared to the White race group, by those 0–17 years, 18–39 years, and 40–64 years compared to those 65+, by females compared to males, and visits using Medicaid/SCHIP/CHIP compared to visits using private insurance all had lower odds of receiving imaging. Further, visits in the West and Northeast, as compared to the South and those in rural areas as compared to urban areas, had lower odds of receiving imaging. Visits by individuals with more chronic conditions were found to have higher odds of receiving imaging. After bottoming in 2014, imaging use increased.

**Conclusion:**

Disparities in the use of imaging for ED patients with a suspected TBI persist. Ongoing public health education efforts are needed to ensure proper screening during ED visits with a suspected TBI and for care post-TBI.

## Introduction

Traumatic brain injury (TBI) results from exposure to an external physical force that disrupts brain function (1). Mechanisms of injury include motor vehicle collisions, falls, penetrating injuries, or polytrauma (2). The Glasgow Coma Scale (GCS) scores determine the level of consciousness and TBI severity, which can range from mild (GCS score 14–15) to moderate (GCS score 9–12) or severe (CGS score 3–8) (2–4). The diagnosis and management of TBIs rely on neuroimaging, and abnormalities such as intracranial injury, mass effect, or cerebral edema (2, 5–7). Computerized tomography (CT) imaging is a standard modality used in the emergency department (ED) to diagnose suspected TBIs (8). Magnetic Resonance Imaging (MRI) can be used to diagnose a more severe type of intracranial injury such as diffuse axonal injuries (DAI) where brain damage is more extensive (5). MRI has been linked to early diagnosis of the severity of the brain injury and the functional outcome and short-term prognosis which can be useful for hospital discharge planning (5). Although guidelines for neuroimaging administration for adults and the pediatric population provide a logical approach to improve outcomes, they have insufficient evidence to support specific recommendations to the point that the brain trauma community are urged to identify research that can address those gaps in the guidelines (6, 7). But one thing that is evident for clinical guidance is that a CT of the head can identify intracranial hemorrhage, contusion, swelling, herniation and compressed basal cisterns that can lead to further neurosurgical evaluation. An MRI provides more sensitive data for cortical contusions and axonal injuries (6, 7). However, abnormalities in patients with mild TBIs (mTBIs) may be missed, never reported, or may only be detected in the minority of patients (10%) who receive a CT scan (9, 10). Therefore, access to imaging and early detection of brain trauma using neuroimaging plays a critical role in classification of head injury, prognosis, and management of TBI outcomes despite patients’ age at the time of injury and the severity brain trauma.

The global incidence of all-cause and all-severity TBI is 939 cases per 100,000 people, with an estimate of between 64 and 74 million new cases of TBI worldwide each year. In high-income countries alone TBIs represent 18.3 million health care cases annually (9). However, mTBIs may occur more frequently (81%) than moderate or severe TBIs but may never be reported (9). The highest frequencies of TBI ED visits, hospitalizations, and deaths are observed among older adults aged ≥75 years, children between 0 and 4 years, and adolescents/young adults between 15 and 24 years (11). These totals only account for those who have received a diagnosis. Conversely, in a prior study that estimated rates of ED visits among older adults, females 65 years of age and older had the highest rates for diagnosed mTBIs (12). Given the high incidence, TBI is a substantial global public health problem that needs attention (2, 13). Promoting prompt access to neuroimaging and giving families a chance to prepare and plan for the unfavorable outcomes of patients who present to the ED with a suspected TBI can offer some post-acute care guidance and serve as an early indicator of prognosis (4, 5).

Unaccounted cases of TBIs, disparities, and inequalities related to access to care exist in the diagnosis and management of TBIs by age, race, ethnicity, sex, socioeconomic status, and geographic location. Previous studies have examined differences in the diagnosis and management of TBI in the ED due to racism, low socioeconomic status, or geographical barriers (11, 14). Other studies have found disparities in health care access in rural regions with fewer resources and the increased incidence of mTBIs in rural locations compared to urban settings (14); lack of consistent access to prehospital care, intracranial pressure monitoring, or intensive care unit admission (15); disparities by race and insurance status on the timely utilization of imaging, treatment, and outcome (16); and the potential lack of accurate injury reporting in regions with fewer resources and high disease burden (9). Additionally, racial and ethnic care disparities due to longer wait time to see a physician because of language barriers and provider-patient misconceptions have also been reported in prior studies (17). Prior research findings and CT utilization trends in the ED between 2007–2017 highlight imaging underutilization and disparities in head-only CT use by age, race/ethnicity, insurance status, and urban/rural location (16).

Given the neuroimaging underutilization and disparities identified in the literature, this study sought to investigate whether disparities in the use of imaging for patients who present to the ED with a suspected TBI are evident in the National Hospital Ambulatory Medical Care Survey. Our study differs from prior research as we aim to describe nationwide ED CT and MRI utilization for suspected TBI visits. We focused on disparities of neuroimaging use (CT scan and MRI) for the potential prognostic value for patients who present to the ED with a suspected TBI, rather than CT only as other studies have described. The results may shed light on opportunities to improve acute and post-acute care given the critical function neuroimaging has on early diagnosis, prognosis, management, and care of individuals who have experienced head trauma. Additionally, results may aid in impacting early predictors of functional outcomes as having access to neuroimaging can be useful for health care providers, patients, and families when making care decisions post-TBI (5).

## Methods

### Study design and setting

This is a retrospective, observational, cross-sectional study of data collected in the National Hospital Ambulatory Medical Care Survey (NHAMCS) for the survey years 2010–2021. This choice of study design was limited by the nature of the NHAMCS – a survey of yearly serial snapshots of visits that occurred in a calendar year. NHAMCS is an annual, national probability sample of ambulatory visits made to non-federal general, and short-stay hospitals in the U. S. conducted by the National Center for Health Statistics (NCHS). Although the survey included visits to hospital outpatient departments from 1992 to 2017, this analysis focuses solely on visits to hospital EDs. The survey has been conducted annually since 1992. The sample design includes three stages for the ED component: (1) 112 geographic primary sampling units that comprise a probability subsample of primary sampling units from the 1985 to 1994 National Health Interview Surveys; (2) approximately 450–500 hospitals within primary sampling units (number varies by survey year); and (3) patient visits within all of the emergency service areas within sampled EDs. In addition, in 2012, a supplemental sample was added to produce estimates for the five largest states. Sample hospitals are randomly assigned to 16 panels that rotate across 4-week reporting periods so that each hospital is surveyed about once every 15 months. The initial sample frame of hospitals was based on the 1991 SMG hospital database now maintained by IQVIA (18, 19).

### Data collection and processing

Hospitals are inducted into NHAMCS by field representatives of the U. S. Census Bureau. From 1992 to 2011, NHAMCS used paper Patient Record forms (PRFs), and data collection was carried out by hospital staff and Census Bureau field representatives. Starting in 2012, NHAMCS switched to an automated mode of data collection, and, by 2016, all data collection was performed by Census Bureau field representatives who abstracted data from medical records for each sampled visit. Data are collected on patient demographics, reasons for visit, vital signs, causes of injury, diagnoses, diagnostic tests ordered or provided, procedures provided, medications given in the ED or prescribed at discharge, providers seen, and visit disposition including hospital discharge information if admitted (since 2005). NHAMCS is approved annually by the NCHS Ethics Review Board with waivers of the requirements to obtain informed consent of patients and patient authorization for release of patient medical record data by health care providers. Medical coding of verbatim text entries for patients’ reasons for visit, causes of injury, medications (through 2011), and providers’ diagnoses is performed by contracted medical coders. Since 2012, medication coding and adjudication has been performed by NCHS. As part of the quality assurance procedure, a quality control sample of PRFs is independently keyed and coded. Error rates typically range between 0.1 and 1.5% for various survey items (18).

The inclusion criteria for this study encompassed all participant visits with suspected traumatic brain injury (TBI) recorded in the NHAMCS-ED datasets from 2010 to 2021. Suspected TBI was operationalized using Reason for Visit (RFV) codes, which were analyzed to reflect screening for TBI. RFV codes were chosen over ICD codes to better capture both the screening process and injury status, aligning with the study’s objectives. This approach is supported by the American College of Radiology guidelines, given the common mechanisms of head trauma—such as falls, motor vehicle accidents, and acts of violence (20).

The presence of a suspected TBI was determined by the reason for visit to the ED codes which could result in a TBI utilizing the variables RFV1-RFV5: 1210.0-headache, pain in head, includes post-traumatic, 3240.0-other special examination, includes neurological exam, 5005.0-fractures and dislocations: head and face, 5205.0-lacerations and cuts: head and neck area, 5305.0-puncture wounds: head, neck, and facial area, 5405.0-contusions, abrasions, bruises: head, neck, and face, 5505.0-injury, other, unspecified type: head, neck, and face, 5805.0-motor vehicle accident, 5810.0-accident (includes falls), 5815.0-violence.

For eligible visits, information was included on participant gender, race, ethnicity, age, payment type, geographic region, metropolitan statistical area (MSA) and the total number of chronic conditions (TOTCHRON). The total number of chronic conditions variable provides a count number of chronic conditions ranging from 0 to 14, but the specific conditions were not recorded. This variable was utilized as a surrogate for morbidity in the study. The age group was categorized to align with the developmental age groups, trends from the literature, and guidelines for management of TBI with a focus on children and adolescents (ages 0–17), adult (ages 18–39), middle age (ages 40–64), and elderly (ages ≥65).

Race and ethnicity are collected as two separate variables in the NHAMCS database. The survey options provided for race are: White, Black or African American, Asian, Native Hawaiian or Other Pacific Islander, American Indian or Alaska Native, and ‘More than one race reported’. The survey options provided for ethnicity are: ‘Hispanic or Latino’ and ‘Not Hispanic or Latino’. Due to small numbers amongst the least prevalent race groups [Asian (*n* = 775), Native Hawaiian or Other Pacific Islander (*n* = 137), American Indian or Alaska Native (*n* = 273), and ‘More than one race reported’ (*n* = 163)], these groups were combined into a single category of ‘Other’ for all analyses. While possible to combine these variables into a single race/ethnicity variable, due to small cell counts for the ‘Black/Hispanic’ (*n* = 93) and ‘Other/Hispanic’ (*n* = 94) race/ethnicity constructs, authors felt it most descriptive and informative to leave the race and ethnicity variables as separate in all analyses. The endpoint of all analyses was the receipt of imaging (yes/no). While acknowledging the inherent differences between diagnostic modalities, the receipt of imaging was determined by whether, at each ED visit, one or more of the four variables any CT scan, CT scan of the head, any MRI or any imaging was completed as ‘yes’. For all visits at which all four variables were completed as ‘no’, the receipt of imaging variable was coded as ‘no’. Because there were visits with two or more imaging modalities recorded as ‘yes’, it was not possible to create an endpoint variable with more than two levels to attempt to assess the impact of each imaging modality separately.

The collected NHAMCS-ED data were analyzed using the sampled visit weight, which represented the product of the corresponding sampling fractions at each stage in the sample design. The sampling weights were adjusted by the NCHS for survey nonresponse as appropriate within the database, yielding a nonbiased national estimate of visit occurrences, percentages, and characteristics. Consistent with the multi-stage, cluster-sampling methods used in NHAMCS, all analyses were weighted and clustered to extrapolate results to generate average annual US national estimates. That is, the analysis of the survey, as designed, allows for the generation of national average annual ED visit totals for the years 2010–2021 by extrapolation of the survey sample (*n* = 38,075) (21–23).

### Statistical analysis

Survey data were analyzed using the sampled visit weight that is the inverse of the selection probabilities, which in turn is the product of the corresponding sampling probabilities at each stage in the sample design. The sampling weights have been adjusted by NCHS for survey nonresponse within time of year (assuming sufficient response by season), geographic region, urban/rural and ownership designations, yielding an unbiased national estimate of ED visit occurrences, percentages, and characteristics. Because of the complex sample design, sampling errors were determined using the SAS SURVEYFREQ and SURVEYLOGISTIC procedures, which consider the clustered nature of the sample. The appropriate SAS procedure options (NOMCAR and DOMAIN) to address missing data and use of domains to determine accurate variance estimates were implemented in the analyses as recommended by the NCHS (18, 21). The data for analyses was de-identified and cleaned by the CDC prior to release.

A multivariable logistic regression model was constructed to evaluate the predictive value of each independent variable of interest amongst those available in the NHAMCS-ED datasets – including all available predictor variables in the model - on suspected TBI. Odds ratios (ORs) with corresponding 95% confidence intervals (CIs) for each level of each discrete variable included in the model, in comparison to each variable’s reference group, were generated and reported. The variables included in the model were grouped for analysis as shown in Table 1. The NCHS recommends that any variable with a survey estimate based on <30 records, with a > 30% missing data or a relative standard error (RSE) of >30%, be excluded from analyses due to potential unreliability (18). None of these thresholds were crossed by any of the variables. As this was a retrospective, hypothesis generating type of study, no adjustments for multiple comparisons were made. In addition, aligning with current thinking regarding best practices against significance testing from thought leaders in statistics, statistical significance was not reported for any results (24). Further, a focus on the provided confidence intervals is urged to ensure the readers’ awareness of both interval width (narrower being more informative) and location (further from zero indicating increasing importance). All analyses were generated using SAS version 9.4 (25). Due to the nature of the NHAMCS data sources used being de-identified and publicly available, this research was deemed Not Human Subject Research by the Campbell University Institutional Review Board.

**Table 1:** Demographic and participant visit characteristics.

Characteristic	Weighted Frequency^a^ (%) of visits *N* = 38,075^b^
Total Number of Chronic Conditions [mean ± SE; (min, max)]	0.9 ± 0.03 (0, 12)
Age Group (years) [mean ± SE]	38.1 ± 0.36
40–64	5,342,318 (26.8)
18–39	6,586,565 (33.1)
0–17	4,399,832 (22.1)
65+	3,570,526 (17.9)
Gender	
Female	10,980,422 (55.2)
Male	8,918,819 (44.8)
Race group	
Other^c^	583,688 (3.6)
Black	3,931,386 (23.7)
White	12,082,642 (72.8)
Ethnicity	
Hispanic/Latino	2,440,940 (15.4)
Non-Hispanic/Latino	13,421,451 (84.6)
Insurance type	
Other^d^	3,083,440 (17.1)
Medicaid/SCHIP/CHIP	5,629,482 (31.3)
Medicare	3,768,091 (21.0)
Private	5,501,799 (30.6)
Region	
West	4,059,553 (20.4)
Midwest	4,570,874 (23.0)
Northeast	3,292,738 (16.5)
South	7,976,076 (40.1)
Urbanicity	
Rural (non-MSA)	2,872,877 (15.6)
Urban (MSA)	15,564,031 (84.4)
Imaging received	
Yes	12,365,661 (62.1)
No	7,533,580 (37.9)

SE, Standard Error; SCHIP, State Children’s Health Insurance Program; CHIP, Children’s Health Insurance Program; MSA, Metropolitan Statistical Area. ^a^Survey weighting, stratification, and clustering accounted for reflecting unbiased, national annual estimates of visit occurrences for the portion of the population meeting the study inclusion and exclusion criteria. ^b^Raw, unweighted survey sample size from which the weighted estimates included in this table were extrapolated. This survey sample size extrapolates to an average, annual estimated total of 19,899,241 emergency room visits. If any counts do not sum to the unweighted total, it is due to missing data. ^c^“Other” includes Asian, Native Hawaiian/Other Pacific Islander, American Indian and Alaska Native, and “More than one race reported”. ^d^“Other” includes workers’ compensation, self-pay, no charge/charity and other.

## Results

The total number of patient visits in the NHAMCS-ED between 2010 and 2021 was 272,170. Of these visits, a total of 38,075 met the study inclusion/exclusion criteria and were included in the data analysis. The mean [standard error (SE)] age was 38.1 (0.36) years. The mean (SE) total number of chronic conditions was 0.9 (0.03) with a minimum of 0 and a maximum of 12. Most of the patient visits were by White (72.8%), Non-Hispanic/Latinos (84.6%), between the ages 18 and 39 years (33.1%) and by females (55.2%). The largest percent of patient visits were covered by Medicaid/SCHIP/CHIP (31.3%) or private insurance (30.6%). The majority of the patient visits occurred in a Metropolitan Statistical Area (MSA) (84.4%) and in the South (40.1%). Overall, 62.1% of patient visits received imaging in the ED for a suspected TBI. Patient visit characteristics are summarized in Table 1.

**Figure 1:**
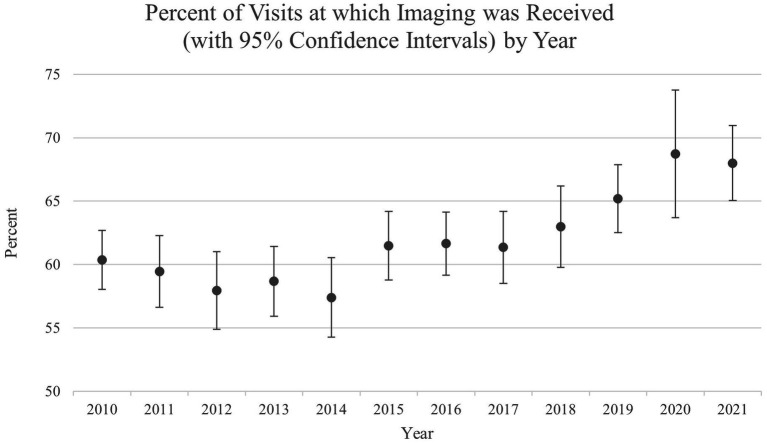
Plot of the annual percentage of visits for the receipt of imaging.

Figure 1 shows the annual percentage of visits at which imaging was received, along with corresponding 95% confidence intervals (CIs). Following a small but fairly steady decline in the percentage of visits at which imaging was received between 2010 and 2014, after bottoming in 2014, there appears to have been a gradual increase in the use of imaging since. When compared to the years with the lowest percentage of visits at which imaging was received (2012–2014), only the most recent years (2019–2021) show any suggestion of an important increase in the use of imaging (Figure 1).

In the multivariable logistic regression model, after adjusting for the other predictor variables in the model, patient visits by the Black race group as compared to the White race group [Odds Ratio (OR) 0.70; 95% CI 0.62–0.80] had lower odds of receiving imaging. When compared to patient visits by those ≥65 years, patient visits by those 0–17 years (OR 0.16; 95% CI 0.12–0.21), 18–39 years (OR 0.28; 95% CI 0.22–0.35), and 40–64 years (OR 0.42; 95% CI 0.34–0.51) had lower odds of receiving imaging. Patient visits by females, as compared to males (OR 0.89; 95% CI 0.80–0.99), had lower odds of receiving imaging as were patient visits by those with Medicaid/SCHIP/CHIP (OR 0.84; 95% CI 0.75–0.95) as compared to patient visits by those with private insurance. Patient visits that occurred in a non-MSA as compared to visits that occurred in a MSA (OR 0.77; 95% CI 0.65–0.91) and in both the West (OR 0.69; 95% CI 0.57–0.84) and Northeast (OR 0.77; 95% CI 0.64–0.93), as compared to visits occurring in the South were all found to have lower odds of receiving imaging. Patient visits with an additional chronic condition were found to have 15% higher odds (95% CI 1.10–1.20) of receiving imaging for a suspected TBI (Table 2).

**Table 2:** Multivariable logistic regression model for receipt of imaging^a^.

Variable^b^	Odds ratio (95% CI)
Ethnicity	
Hispanic or Latino vs. Not Hispanic or Latino	0.97 (0.81, 1.16)
Race	
Other^c^ vs. White	0.85 (0.63, 1.16)
Black vs. White	0.70 (0.62, 0.80)
Age Group	
40–64 vs. 65+	0.42 (0.34, 0.51)
18–39 vs. 65+	0.28 (0.22, 0.35)
0–17 vs. 65+	0.16 (0.12, 0.21)
Gender	
Female vs. Male	0.89 (0.80, 0.99)
Insurance Type	
Other^d^ vs. Private	1.00 (0.87, 1.15)
Medicaid/SCHIP/CHIP vs. Private	0.84 (0.75, 0.95)
Medicare vs. Private	0.95 (0.79, 1.16)
Region	
West vs. South	0.70 (0.58, 0.85)
Midwest vs. South	0.89 (0.76, 1.03)
Northeast vs. South	0.77 (0.65, 0.91)
Urbanicity	
Non-MSA vs. MSA	0.77 (0.65, 0.91)
Total number of chronic conditions	1.15 (1.10, 1.20)

SCHIP, State Children’s Health Insurance Program; CHIP, Children’s Health Insurance Program; MSA, Metropolitan Statistical Area; CI, Confidence Interval. ^a^Survey weighting, stratification, and clustering accounted for reflecting unbiased, national annual estimates of visit occurrences for the portion of the population meeting the study inclusion and exclusion criteria. ^b^The reference level is listed last in each row. ^c^“Other” includes Asian, Native Hawaiian/Other Pacific Islander, American Indian and Alaska Native, and “More than one race reported.” ^d^“Other” includes workers’ compensation, self-pay, no charge/charity and other.

With a large, nationally-representative dataset, the results of this study suggest disparities in imaging use for suspected TBI across demographic, geographic, and insurance-related factors. Patient visits by those in the Black race group (compared to those in the White race group), those in younger age groups, those with Medicaid/SCHIP/CHIP (compared to those with private insurance), those living in West and Northeast (compared to the South), those visiting an ED in rural areas and females had lower odds of receiving imaging. Contrarily, those with a higher number of chronic conditions had increased odds of receiving imaging. A clear imaging underutilization was noted from 2010 to 2014; but between 2019 and 2021, a gradual increase was seen which may reflect changes in ED protocols.

## Discussion

This paper sought to investigate the presence of disparities in the use of imaging including CT and MRI usage for patients who present to the ED with a suspected TBI utilizing the most recent data collected in the NHAMCS for the survey years 2010–2021. Findings from this research were consistent with the authors’ hypothesis and predictions, indicating potentially meaningful differences in imaging use between different racial groups, age groups, genders, insurance type, geographic region, urbanicity and the total number of chronic conditions a patient had at the time of their ED visit. However, it is important to clarify that CT scans and MRI are distinct imaging modalities with different diagnostic capabilities and not interchangeable neuroimaging tools in the clinic considering the cost-effectiveness or sensitivity approaches needed to evaluate the severity of the brain trauma (6, 7). In this regard, caution is urged in the interpretation of the results of this study since of the 22,999 visits with imaging included in this study, only 520 (approximately 2.3%) involved MRI. As mentioned in the Methods section, this forced the authors to combine CT and MRI visits into a catch-all of ‘any imaging performed’ to obtain a more robust and interpretable assessment of imaging utilization patterns and potential disparities in this patient population.

Visits to the ED by those in the Black race group were estimated to have 30% lower odds of receiving imaging after a suspected TBI compared to White individuals. However, no meaningful disparities in the utilization of imaging in the ED amongst ethnic groups was found. Furthermore, patient visits with Medicaid/SCHIP/CHIP had 16% lower odds than those with private insurance, indicating disparities in imaging utilization. A recent study published in 2022 analyzed CT usage (any CT or CT head) in the ED setting for the years 2007–2017 using NHAMCS data utilizing similar reasons for visit as our study (16). However, the current study has a narrower focus specific to suspected TBIs. Our results support their findings in the reduced use of CT for visits by non-Hispanic Black, younger, and Medicaid patients despite the clinical need for imaging for diagnosis. Our study results add data related to MRI use which plays a critical role in the early diagnosis of the severity of the brain injury which can be fundamental for cautious early prognosis in functional outcomes post-injury. Renson et al. research findings also indicated that uninsured other non-Hispanics (including multiracial) were 24% less likely to receive a CT scan; non-Hispanic White at 12% less likely, and non-Hispanic Black 18% less likely compared to their insured counterparts utilizing data from the National Trauma Data Bank from 2010 to 2015 (26).

The observed difference between the Black and White race groups may be explained by socioeconomic status, as those in underrepresented or minority groups may lack adequate health insurance coverage and timely access to care (27, 28). Other studies have previously reported longer wait times between ED presentation and head imaging performance for minorities, stating that Black patients, on average, waited about 8 min longer than non-Black patients (29). While the current study did not include wait time in the analysis, any such difference between racial groups may exacerbate the observed disparities and the potential implicit bias which may impact rehabilitation timing and access to ancillary services for specific outcomes post brain injury (30).

Some studies suggest that lack of neuroimaging use and the differences between the ethnic groups could be due to infrastructure barriers such as reduced resources, medical setting availability, reduced neuroimaging in rural hospitals, or care barriers such as language differences and implicit bias (29, 31). Inadequate access to Spanish interpreters may cause delayed interaction between patient and provider, which subsequently can impact the timely diagnosis and care of Hispanic/Latino patients. The current study’s utilization of the most recently available NHAMCS data and the lack of evidence of any disparity amongst the ethnic groups leaves the authors hopeful that the issues pointed out by the Alter et al. and Yue et al. papers may be diminishing. Further, the focus of the present study on suspected TBIs in the ED may result in less bias in comparison to imaging in a non-emergency setting given the potential critical nature of such injuries and necessary immediacy of treatment. The objective data that CTs and MRIs provide can lead to optimal and timely assessment, intervention, and rehabilitation of individuals who experience a TBI.

Our study found that older adults (≥ 65 years) were found to be more likely to receive imaging than those under 65. Visits to the ED for a suspect TBI by those 0–17, 18–39 and 40–64 year of age were estimated to have 84, 72 and 58% lower odds of receiving imaging, respectively, as compared to those 65+. In addition, for each additional chronic condition, the odds of receiving imaging at an ED visit for a suspected TBI were shown to increase by 15% on average. Not surprisingly and according to past studies, individuals aged 65 and older with a TBI have been shown to have pre-existing conditions such as diabetes, cardiovascular disease, pulmonary disease, or renal disease and a higher mortality rate (32); and as the number of chronic conditions increases, the health quality outcomes for the individual are reduced (33). Differences in the use of imaging for older patients may be also attributed to increased fragility, risk for falls, and mortality risks in older adults compared to younger adults (30, 33, 34). It can also be supported by specific age-related guidelines without loss of consciousness and suspected TBI (35), and medications associated with early hemorrhage (36).

This study found gender-based differences in the receipt of imaging for visits to the ED for suspected TBIs, with visits by females having 11% lower odds of receiving imaging as compared to males. However, the reason behind this disparity remains unclear. Possible speculative explanations may be attributed to differences in symptom perception and reporting (30), age (12), and underreporting due to the fear women experience after intimate partner violence is evident, as 1 in 10 women with injuries localized to the head fail to seek medical attention out of fear of being stopped by the partners (37). Therefore although not directly supported by the study data, the gender disparity results from this study could suggest a potential connection with psychosocial reasons for females who present to the ED with a suspected head injury that needs to be further investigated (38).

Inadequate information regarding symptom presentation can cause a knowledge gap in the diagnosis and care of females who present to the ED with a suspected TBI and varying complications for health outcomes up to a year post-head trauma (30, 31). According to the literature, females, who comprise almost 20% of all TBIs (293/1627), have a higher mortality (3.4%) resulting from TBIs, when compared to males (1.6%) (13). Other studies have reported up to 50.8% of mTBI visits by female patients (39). Further, mTBIs have been linked to severe complications such as intracranial hemorrhage (14) and incomplete recovery for females (RR 1.31; 95% CI 1.15–1.49) diagnosed with mTBI as compared to males (40). Hence the importance of imaging use for screening in the ED to establish if any brain abnormalities are present and to determine the functional prognosis in female patients is paramount.

This study demonstrated that disparities in imaging may exist based on geography. Specifically, those with an ED visit for a suspected TBI in a rural area (as compared to an urban area) as well as those with a visit in the Northeast and West (as compared to the South) had lower odds of receiving imaging. These findings align with previous research highlighting lower imaging use and accessibility disparities in rural areas often due to limited resources, the absence of standardized TBI management protocols, and increase in fatality rates in such areas (41). Similarly, Daugherty et al. found that areas with a higher proportion of patients living in rural areas and the South had higher rates of TBI-related deaths, whereas patient visits in the northeast and those not living in rural areas have lower TBI-related deaths (42). This potential association between reduced imaging use and higher fatality rates needs to be investigated further.

Our research findings demonstrate an overall increased trend in imaging utilization between 2010 and 2021. This increase in imaging utilization for ED visits with a suspected TBI between 2014 and 2020 may be related to clinician factors such avoiding malpractice issues or systemic and environmental factors such as insurance or records transferability (43). In contrast, Dubey et al. reported no significant change in head CT utilization rate between 2007 and 2017 (4% change). An increase in imaging as the incidence of TBI’s increases would be expected. This increase in imaging utilization may also be attributed to technological advancements, increased demand by patient visits or physicians, and increased revenue (44). However, given the findings of the current study regarding potential imaging disparities based on race, gender, and geography region and the prevention awareness we seek to convey, the use of imaging seems crucial for identification, prognosis, and rehabilitation management given the challenges and mortality risks that individuals who have experienced TBIs may have (31).

According to the updated TBI monitoring recommendations published in 2016, intracranial pressure monitoring is recommended to reduce in-hospital and two-week post injury mortality for all patients with a GCS 3–8 (severe TBI) and an abnormal CT scan (7). However, with the introduction of Abbott’s rapid i-STAT TBI plasma test beginning in 2021 which assists clinicians in assessing patients with a suspected TBI (45), and the campaigns created to avoiding unnecessary testing such as “choosing wisely,” (46) a decrease in imaging frequency over time is to be expected subsequent to the years included in the current study. Any such observed decrease will likely depend on the adoption of TBI screening in the clinical setting and public health initiatives created to increase the percentage of adults who resume normal participation after a TBI as well as rehabilitation services to accelerate progress (30, 45–47). Further, the methods used for TBI screening and under or overutilization of neuroimaging may also depend on various multidimensional factors and supporting structures that influence the provider’s decision-making process in determining whether or not to use neuroimaging such as personal experience, professional judgement, professional responsibility, consultation with ED staff or any legal repercussions (48). Therefore, future research is warranted to more thoroughly examine the elements that impact providers choices in hospital emergency services and the effect of Abbott’s rapid i-STAT TBI plasma on any imaging trend.

## Conclusion

Disparities in the use of imaging for patient visits to the ED with a suspected TBI remain, including various factors such as race, age, sex, geographic region, insurance payment type, Metropolitan Statistical Area (MSA), and the total number of chronic conditions. Even though most patient visits to the ED for a suspected TBI receive imaging (62%) and an overall increase in the use of imaging was seen in our results, some patient visits remain less likely to receive imaging. To understand the extent of the problem more fully, future research should be conducted to assess underuse vs. overuse across these disparities. In the meantime, opportunities currently exist to improve the diagnosis and management of emergency care for individuals who have suffered TBI to improve best practices as well as outcomes. Lastly, given the increase incidence of geriatric TBI, further research is needed to measure older adult frailty, multiple drug intake, and comorbidities to predict functional outcomes post injury using imaging. Consequently, ongoing efforts to address the disparities in the use of imaging for patient visits that present to the ED with a suspected TBI found in this study should be a public health priority that can be addressed through awareness training and the above mentioned strategies using findings from our research.

### Recommendations for readers

The evaluation of patients with a suspected TBI is complex. Guidelines for the management of TBIs exist, however they do not constitute a complete protocol to guide providers’ decision-making. Furthermore, disparities in the use of imaging continue to be evident, perhaps due to the lack of clarity in aspects of clinical practice that have not been supported by research. Therefore, the recommendations from the current study emphasize the benefits of individualized care and the value of neuroimaging in early prognostics. The relative importance of neuroimaging and the opportunities it can offer to increase public awareness, provide prompt post-acute care and offer information about hospital discharge recommendations to families who need to make difficult decisions is valid for the authors of this study. For example, an occupational therapy practitioner or allied health care professional who has access to neuroimaging and provider notes can educate families on expected functional status, environmental modification needs, and discharge recommendations that may help them understand recovery or sequelae for individuals who have experienced head trauma.

### Strengths and limitations

This study boasts a large sample size utilizing nationally representative survey data over an 11 years’ timeframe. However, there were database limitations. Variables included in the analyses were restricted by the data available in the NHAMCS-ED survey. The RFVs were selected based on the authors’ clinical assessment of what could lead to a suspected TBI and available codes that captured both the TBI screening process and injury status due to common mechanisms of head trauma such as falls, motor vehicle accidents, and acts of violence. Some were general while others were more specific. This could lead to potential selection bias in the current study population, but RFV was the only way to capture suspected TBI (which was the intent of the study) rather than diagnosis. The appropriateness of indications for imaging was not assessed due to the absence of that information in the survey data. The study endpoint of imaging received was considered as positive if any one or more of the variables CAT scan, head CT, brain MRI or ‘any imaging’ was responded to as ‘yes’ based on the literature review and RFVs utilized for the suspected TBI. While it is widely agreed that CT imaging and MRI are not interchangeable in the clinic, due to the limited number of visits with an MRI in this study as well as many visits having more than one imaging modality coded as ‘yes’, these dataset limitations did not allow for analyzing CT imaging and MRIs separately. Further, there is a possibility of information bias in the imaging definitions used in this study. However, the authors opted for a broader definition rather than limiting it to head imaging alone. This decision was influenced by the inclusion of general RFV codes such as accidents (falls), motor vehicle accidents, and violence, which would have required comprehensive imaging of the entire body given the anticipated multiple body injuries upon ED arrival. In addition, the database does not include data on severity of injuries nor intentionality, therefore, those factors were not included in the study. Furthermore, the survey is conducted cross-sectionally so no longitudinal type analyses (e.g., repeated head injuries) are possible. Finally, it is important to note that the NHAMCS survey uses physician-based reporting to collect data, helping to reduce/prevent bias found in self-reported patient surveys.

## Data Availability

Publicly available datasets were analyzed in this study. This data can be found at: https://www.cdc.gov/nchs/nhamcs/documentation/index.html.
